# Excitonic effects on the second-order nonlinear optical properties of semi-spherical quantum dots

**DOI:** 10.1186/1556-276X-6-268

**Published:** 2011-03-29

**Authors:** Jefferson Flórez, Ángela Camacho

**Affiliations:** 1 Departamento de Física, Universidad de los Andes, A.A. 4976, Bogotá, DC, Colombia

## Abstract

We study the excitonic effects on the second-order nonlinear optical properties of semi-spherical quantum dots considering, on the same footing, the confinement potential of the electron-hole pair and the Coulomb interaction between them. The exciton is confined in a semi-spherical geometry by means of a three-dimensional semi-parabolic potential. We calculate the optical rectification and second harmonic generation coefficients for two different values of the confinement frequency based on the numerically computed energies and wavefunctions of the exciton. We present the results as a function of the incident photon energy for GaAs/AlGaAs quantum dots ranging from few nanometers to tens of nanometers. We find that the second-order nonlinear coefficients exhibit not only a blue-shift of the order of meV but also a change of intensity compared with the results obtained ignoring the Coulomb interaction in the so-called strong-confinement limit.

## Introduction

Nonlinear optical properties of semiconductor quantum dots have attracted considerable interest due to their several potential applications [1-4]. In particular, second-order nonlinear optical properties, such as nonlinear optical rectification (OR) and second harmonic generation (SHG), have received special theoretical [5-8] and experimental [9,10] attention due to their magnitudes being stronger than those of high-order ones, making them the first nonlinear optical effects experimentally observed.

The confinement of carriers provided by a quantum dot is well described by a parabolic potential when only the lowest excited states of the carriers are considered. However, self-assembled quantum dots growth in the laboratory usually exhibit asymmetrical shapes that ensure the generation of nonlinear optical effects. In order to model these asymmetries, an asymmetrical potential is required.

Recently, several authors [5,6,8] studied the effects of an exciton on the second-order nonlinear properties in one-dimensional semi-parabolic quantum dots. Using analytical approximate results, they showed that the excitonic effects enhance significantly the OR and SHG coefficients. They used the so-called strong-confinement limit, ignoring in this way the Coulomb interaction between electron and hole because of the quantum dot dimensions are smaller than the effective Bohr radius, and finding that the excitonic effect reduces itself to an effective-mass model in one particle scheme.

In this study we find eigenenergies and eigenstates of an exciton in a semi-spherical quantum dot solving the corresponding three-dimensional Schrödinger equation using a finite elements method and taking into account both the confinement and Coulomb potentials of the electron-hole pair. We present the OR and SHG coefficients as a function of the incident photon energy with and without Coulomb potential. Our results show that energy and intensity of the peaks in the second-order nonlinear optical coefficients change when Coulomb interaction is introduced.

This article is organized as follows. In "Theory" section, we present the characteristic quantities of the harmonic and Coulomb potentials, and the definitions of the weak- and strong-confinement limits in terms of these parameters. In addition, we present the analytical expressions for the optical nonlinearities, such as OR and SHG, obtained by the density matrix formalism. In "Results" section, we show the OR and SHG coefficients with and without Coulomb interaction as a function of the incident photon energy for two quantum dot sizes. We also give account of the changes presented by the second-order nonlinear coefficients focusing in the role played by the Coulomb interaction. Conclusions are summarized in final section.

## Theory

The effective-mass Hamiltonian for the electron-hole pair in the three-dimensional quantum dot reads [11](1)

where  and  are the effective masses of the electron and the hole, respectively, *ε *is the background dielectric constant and *V *(**r***_i_*) is the three-dimensional semi-parabolic potential that we define as(2)

The angle *θ *is the usual polar angle in spherical coordinates, and *ω*_0 _the oscillator frequency considered in this study the same for the electron and the hole. The potential defined in Equation 2 confines the exciton in the upper half of a sphere, i.e., the quantum dot has a semi-spherical shape.

Hamiltonian (1) can be separated in terms of center-of-mass and relative coordinates, respectively,(3)

where  is the total mass, and  is the reduced mass. The center-of-mass and relative position coordinates are defined as usual,(4)

with the corresponding momenta **P **= - *iħ***∇_R _**and **p **= - *iħ***∇_r _**in terms of **p**_e _and **p**_h_,(5)

The explicit separability of the center-of-mass and relative coordinates in Equation 3 lead to the following total envelope wave function and total energy for the system:(6)(7)

The center-of-mass part of Hamiltonian (3) is a three-dimensional semi-parabolic oscillator that can be solved analytically. Therefore, the problem is now reduced to solve the relative motion Hamiltonian:(8)

Hamiltonian (8) has been solved analytically in two limiting cases (strong and weak confinement) for one-dimensional quantum dots. The eigenfunctions and eigenvalues are presented in references [5] and [8]. In one-dimensional case, the confinement potential also imposes constraints to spatial coordinates, resulting in a hydrogen-like (asymmetric-harmonic) reduced particle Hamiltonian for weak (strong) limit.

The harmonic potential in Equation 8 defines both the size *L *of the quantum dot,(9)

and the energy quanta *ħω*_0 _due to confinement, which is related to *L *by(10)

On the other hand, the Coulomb potential defines the effective Bohr radius  and the effective Rydberg energy  of the electron-hole interaction,(11)(12)

The strong-confinement limit is established when , or equivalently , and the weak-confinement limit when , or .

The second-order nonlinear optical coefficients can be obtained by density matrix approach and perturbation expansion method [12,13]. The expression for the OR coefficient, within a two-level system approach, is given by [5,6](13)

where *e *is the electron charge, σ_s _is the density of electrons in the quantum dot, *T*_1 _is the longitudinal relaxation time, *T*_2 _is the transverse relaxation time, and(14)(15)(16)

For the resonance condition(17)

there is a peak intensity given by (1/*T*_1 _≪ 1/*T*_2 _≪ *ω*_01_)(18)

The SHG coefficient in a three level system is [8](19)

where *N *is the density of carriers in the quantum dot, *E_ij _*= *E_i _*- *E_j_*, *Γ*_10 _= *Γ*_20 _= *Γ*_0 _are the relaxtion rates, and(20)

Under the double resonance condition, i.e., *ħω *≈ *E*_10 _≈ *E*_20_/2, the intensity of the peak is given by(21)

and its energy by(22)

## Results

In this study, the results are presented for a GaAs/AlGaAs structure. We have used the following parameters in the calculations:  = 0.067*m*_0_,  = 0.09*m*_0 _(*m*_0 _is the mass of a free electron) [4], *T*_1 _= 1 ps, *T*_2 _= 0.2 ps [12], σ_s _= 5 × 10^24 ^m ^-3 ^[5], *ε *= 12.53, *Γ*_0 _= 1/0.14ps ^-1^, *N *= 3 × 10^16 ^cm^-3 ^[8].

**Figure 1:**
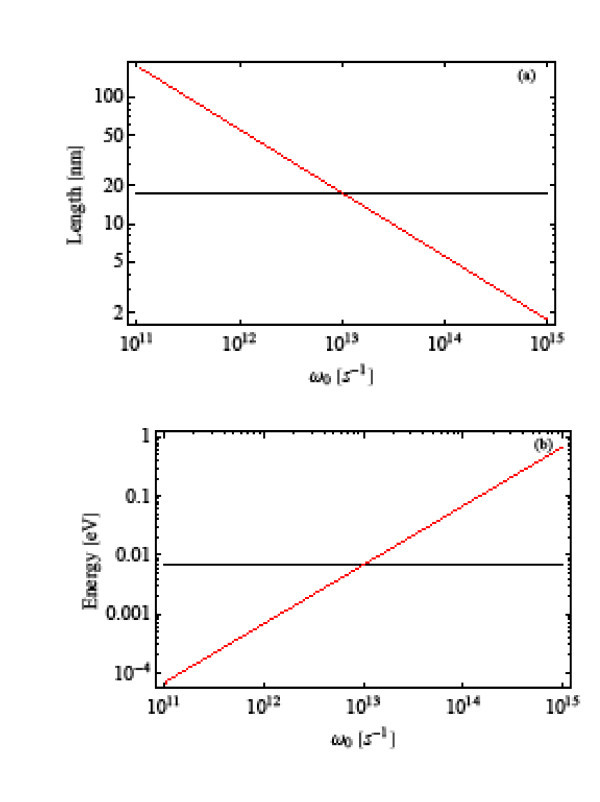
**Characteristic (a) lengths and (b) energies for the confined particle in a GaAs/AlGaAs quantum dot as a function of the confinement frequency**. The red (black) lines correspond to *L *and *ħω*_0 _( and ), respectively.

In Figure 1, we plot the characteristic lengths and energies for the confined particle in a GaAs/AlGaAs quantum dot as a function of the confinement frequency *ω*_0_.  and  are independent on *ω*_0 _because they are related to the Coulomb potential. In Figure 1a, we can see that the lengths *L *and  are of the same order of magnitude for a confinement frequency around *ω*_0 _= 1 × 10^13 ^s^-1^. In Figure 1b, we observe that also *ħω*_0 _and  show similar values around *ω*_0 _= 1 × 10^13 ^s^-1^. For this reason, we conclude that, in this frequency range, neither the strong-confinement limit nor the weak limit can be assumed because both interactions, harmonic and Coulomb, are important. Therefore, we propose a numerical technique to calculate eigenenergies and eigenstates of Hamiltonian (8), considering the harmonic and Coulomb potentials.

With the aim of exploring the nonlinear behavior at higher frequencies, i.e., when the quantum dot size is smaller than in the previous case, we choose *ω*_0 _= 2 × 10^14 ^s^-1^, in which the quantity *L *is less than , or *ħω*_0 _is greater than , differing in both cases by one order of magnitude as can be seen in Figure 1. Because of this difference, several authors[5,6,8] used the strong-confinement limit as a satisfactory approximation in the case of small quantum dots. Accordingly with Equation 9, the frequencies *ω*_0 _= 1 × 10^13 ^s^-1 ^and *ω*_0 _= 2 × 10^14 ^s^-1 ^define a quantum dot size of *L *= 17.4 nm and *L *= 3.9 nm, respectively. This means that our results are suitable for the current quantum dot sizes that range from few nanometers to tens of nanometers.

We find numerically eigenenergies and eigenstates of Hamiltonian (8) by a finite elements method for the two frequencies mentioned above. We have used the software COM-SOL Multiphysics, which offers the possibility of defining a geometry, in this case the upper half of a sphere, and to solve the time-independent Schrödinger equation with appropriate boundary conditions.

**Figure 2:**
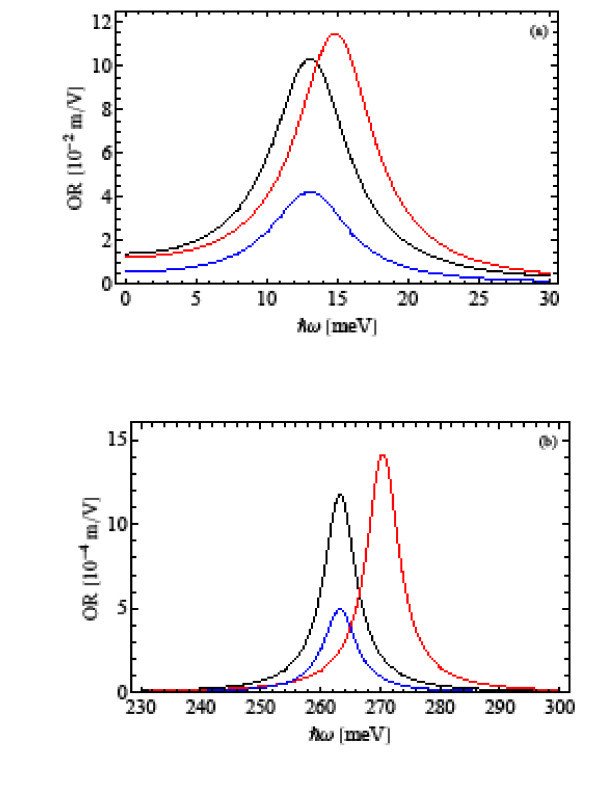
**The OR coefficient as a functions of the incident photon energy *ħω *for (a) *ω*_0 _= 1 × 10^13 ^s^-1 ^and (b) *ω*_0 _= 2 × 10^14 ^s^-1^, considering excitonic effects with (red line) and without Coulomb (black line)interaction**. The blue line corresponds to the case without excitonic effects.

**Figure 3:**
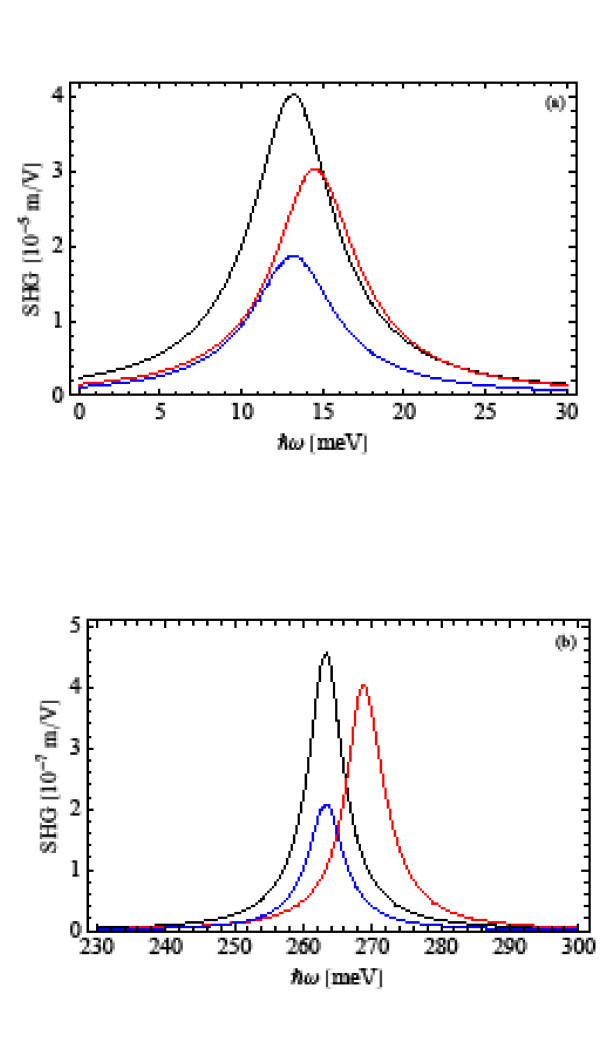
**The SHG coefficient as a functions of the incident photon energy *ħω *for (a) *ω*_0 _= 1 × 10^13 ^s^-1 ^and (b) *ω*_0 _= 2 × 10^14 ^s^-1^, considering excitonic effects with (red line) and without Coulomb (black line)interaction**. The blue line corresponds to the case without excitonic effects.

The terms involving quantum states and energies in Equations 13 and 19 are found using the eigenstates and eigenenergies previously calculated. The OR and SHG coefficients are shown in Figures 2 and 3, respectively. Figures 2a and 3a correspond to *ω*_0 _= 1 × 10^13 ^s^-1^, and Figures 2b and 3b to *ω*_0 _= 2 × 10^14 ^s^-1^. In each figure, we present the corresponding nonlinear optical coefficient considering excitonic effects with and without Coulomb interaction. For comparative purposes, we also present the case without excitonic effects, i.e., when only one electron exists in the quantum dot.

Figures 2 and 3 reproduce the reported results quite well [5,8] regarding the enhancement of the nonlinear optical coefficients due to the quantum confinement of the exciton. However, the same figures show additionally a significant blue-shift of the OR and SHG peaks when both the harmonic and Coulomb potentials are taken into account. In Tables 1 and 2, we present the eigenenergies of the exciton and peak energies of the OR and SHG coefficients with and without Coulomb interaction for the two frequencies under study. The peak energies are estimated by Equations 17 and 22 for the OR and SHG coefficients, respectively.

**Table 1:** Eigenenergies of the exciton and peak energies of the OR and SHG coefficients with and without Coulomb interaction for *ω*_0 _= 1 × 10^13 ^s^-1^

Energy (meV)	With Coulomb	Without Coulomb	Diff.
*E*_0_	11.218	16.455	5.237
*E*_1_	26.147	29.619	3.472
*E*_2_	40.000	42.784	2.784
OR peak energy	14.929	13.164	1.765
SHG peak energy	14.660	13.164	1.496

**Table 2:** Eigenenergies of the exciton and peak energies of the OR and SHG coefficients with and without Coulomb interaction for *ω*_0 _= 2 × 10^14 ^s^-1^

Energy (meV)	With Coulomb	Without Coulomb	Diff.
*E*_0_	306.59	329.10	22.51
*E*_1_	577.06	592.39	15.33
*E*_2_	843.31	855.67	12.36

OR peak energy	270.47	263.28	7.19
SHG peak energy	269.41	263.28	6.13

We can see from Tables 1 and 2 that the eigenenergies obtained with Coulomb inter-action are smaller than those obtained without that interaction. The explanation to this fact is that there is an attractive Coulomb potential between the electron-hole pair that implies a reduction of the eigenenergies for the exciton. However, the eigenenergies are affected in different ways depending on the quantum state. For example, for the ground state *ω*_0 _= 1 × 10^13 ^s^-1^, Table 1, we have an energy difference of 5.237 meV, while for the first and second excited states the differences are of 3.472 and 2.784 meV, respectively. We have a similar situation for *ω*_0 _= 2 × 10^14 ^s^-1^, Table 2. This is because the mean spatial separation between the electron and the hole increases, and therefore the Coulomb interaction decreases, as the energy of the quantum state increases. The final result is a blue-shift of the OR and SHG peaks of the order of meV for both *ω*_0 _= 1 × 10^13 ^s^-1 ^and *ω*_0 _= 2 × 10^14 ^s^-1^.

In addition, the OR and SHG coefficients exhibit different peak intensities depending on the consideration of the Coulomb interaction, as it can be seen in Figures 2 and 3. This fact originates from the modification of the dipole matrix elements defined in Equations14, 15, and 20 when the Coulomb interaction is considered. According to Equation 18, the peak intensity of OR coefficient depends essentially on the product , while for SHG coefficient, Equation 21, the peak intensity depends on *μ*_01 _*μ*_12 _*μ*_20_. Tables 3 and 4 show the values of these dipole matrix element products with and without Coulomb interaction for the two frequencies considered. As one can see, the product  is greater with Coulomb interaction than without it for both confinement frequencies. Therefore, in Figure 2a, b, the OR intensity is higher in the former case than in the later one. In the case of SHG coefficient, the product *μ*_01 _*μ*_12 _*μ*_20 _is smaller with Coulomb than without that interaction. This fact makes the SHG intensity smaller in the former case as can be seen in Figure 3a, b.

**Table 3:** Dipole matrix element products of the OR and SHG coefficients with and without Coulomb interaction for *ω*_0 _= 1 × 10^13 ^s^-1^

Coefficient	[nm^3^]	With Coulomb	Without Coulomb
OR		1365	1222
SHG	*M*_01_*M*_12_*M*_20_	1237	1635

**Table 4:** Dipole matrix element products of the OR and SHG coefficients with and without Coulomb interaction for *ω*_0 _= 2 × 10^14 ^s^-1^

Coefficient	[nm^3^]	With Coulomb	Without Coulomb
OR		16.95	14.03
SHG	*μ*_01_*μ*_12_*μ*_20_	17.65	18.55

## Conclusions

Contrary to the assumption that Coulomb interaction can be neglected when the quantum dot dimensions are smaller than the effective Bohr radius, we show that this interaction affects the excitonic effects of the second-order nonlinear optical properties of semi-spherical quantum dots. We find that Coulomb interaction manifests itself in a blue-shift of the energy peaks of the order of several meV in the studied spectra. These results were found for two quantum dot sizes, in the first one the characteristic quantities of the harmonic and Coulomb potentials are equals, and in the second one they differ by one order of magnitude. This means that the Coulomb interaction plays an important role even when the quantum dot sizes are smaller than the effective Bohr radius.

Therefore, we encourage experimentalists to carry out measurements of second-order optical nonlinearities in asymmetrical quantum dots with the aim of to detect the magnitude of this effect.

## Abbreviations

OR: optical rectification; SHG: second harmonic generation.

## Competing interests

The authors declare that they have no competing interests.

## Authors' contributions

JF: carried out the numerical calculations and drafted the manuscript. AC: performed ananalys and interpretation of results, and gave final approval of the version to be published. All authors read and approved the final manuscript.
